# Longitudinal demographic study of wild populations of African annual killifish

**DOI:** 10.1038/s41598-018-22878-6

**Published:** 2018-03-19

**Authors:** Milan Vrtílek, Jakub Žák, Matej Polačik, Radim Blažek, Martin Reichard

**Affiliations:** 1 Institute of Vertebrate Biology, The Czech Academy of Sciences, Květná 8, Brno, 603 65 Czech Republic; 2 Faculty of Sciences, Charles University, Viničná 7, Praha 2, 128 44 Czech Republic

## Abstract

The natural history of model organisms is often overlooked despite its importance to correctly interpret the outcome of laboratory studies. Ageing is particularly understudied in natural populations. To address this gap, we present lifetime demographic data from wild populations of an annual species, the turquoise killifish, *Nothobranchius furzeri*, a model species in ageing research, and two other species of coexisting annual killifishes. Annual killifish hatch synchronously, have non-overlapping generations, and reproduce daily after reaching sexual maturity. Data from 13 isolated savanna pools in southern Mozambique demonstrate that the pools supporting killifish populations desiccated 1–4 months after their filling, though some pools persisted longer. Declines in population size over the season were stronger than predicted, because they exceeded the effect of steady habitat shrinking on population density that, contrary to the prediction, decreased. Populations of *N. furzeri* also became more female-biased with progressing season suggesting that males had lower survival. *Nothobranchius* community composition did not significantly vary across the season. Our data clearly demonstrate that natural populations of *N. furzeri* and its congeners suffer strong mortality throughout their lives, with apparent selective disappearance (condition-dependent mortality) at the individual level. This represents selective force that can shape the evolution of lifespan, and its variation across populations, beyond the effects of the gradient in habitat persistence.

## Introduction

Model species provide key insights into many aspects of fundamental and applied biological research. However, despite the fact that laboratory conditions often differ considerably from model species’ natural environment, it is usual to interpret the outcome of laboratory studies without the context of the evolutionary and natural history of these species. This is unfortunate because information about the selective forces (environmental or biotic) driving particular adaptations may provide stronger insights into the general understanding of the key findings in laboratory populations^1^.

For a long time, the mere existence of ageing in the wild was regarded negligible and unimportant. Animals were generally considered to succumb to predation or pathogens before they started to age. However, recent evidence demonstrates that ageing is widespread in natural populations^2^. Despite the challenges involved in studying ageing in natural populations, the understanding of the ageing process (demographic, functional and reproductive declines) gained from natural populations is indispensable^3–7^. For example, Warner *et al*.^6^ challenged the classic view by showing that even a long-lived ectotherm (painted turtle, *Chrysemys picta*) can suffer from considerable fitness reduction and elevated mortality in advanced age. To that end, describing the dynamics of demographic parameters in wild populations is crucial to our understanding of how natural selection acts on life-history evolution.

The African turquoise killifish, *Nothobranchius furzeri*, is an established model in several biological disciplines^8,9^, particularly in ageing research^10–12^. *Nothobranchius furzeri* is the shortest-lived vertebrate that can be bred in captivity, with a median lifespan of 2–6 months^9,13^, maximum lifespan of approximately 1 year^14^ and laboratory generation time of 2–4 months^9,15^. These figures are more similar to the life-history schedule of invertebrate models than for a vertebrate^8^. Despite its extremely short lifespan, *N. furzeri* possesses all the hallmarks of vertebrate ageing and its lifespan is condensed rather than terminated prematurely^8^. Over the last decade, exceptional insights have been gained into the biology of ageing in *N. furzeri* and the species is at the forefront of the current fundamental and applied ageing research agenda^8,10–12,16^.

In contrast, very little is known about the ageing of *N. furzeri* in natural populations. The lifespan and ageing of *N. furzeri* in natural populations have never been estimated and are subject to scientific debate. While Terzibasi *et al*.^17^ suggested that mortality in wild populations is primarily caused by habitat desiccation, Reichard *et al*.^18^ demonstrated that it is strongly male-biased and hence likely includes components of sex- and condition-dependence. This is an important difference because theory suggests that the evolution of lifespan and ageing is strongly contingent upon the source of mortality^19^. When mortality is condition-independent (as in the case of habitat desiccation), increased risk of mortality is predicted to select for shorter lifespan and more rapid ageing^20,21^. However, when mortality is condition-dependent and low condition individuals disappear from the population earlier, the predictions regarding selection for lifespan and ageing are more complex^22–24^. Strong condition-dependent mortality could select for high individual condition overall (e.g. large body size, high immunity or social dominance), ultimately leading to extended lifespan and slower ageing in environments where populations are protected from the source of condition-dependent mortality, such as in captivity^25–27^.

In this study, we focus on the demographic parameters of *N. furzeri* populations and their sympatric congeners in the wild. Demographic (actuarial) ageing describes the increase in risk of mortality with increasing age at the population level. A better understanding of the dynamics of mortality in natural conditions will help to identify underlying relationships and selective forces that have led to the evolution of an extremely short lifespan in *N. furzeri* and thus provide data essential for the interpretation of laboratory-based studies.

**Figure 1:**
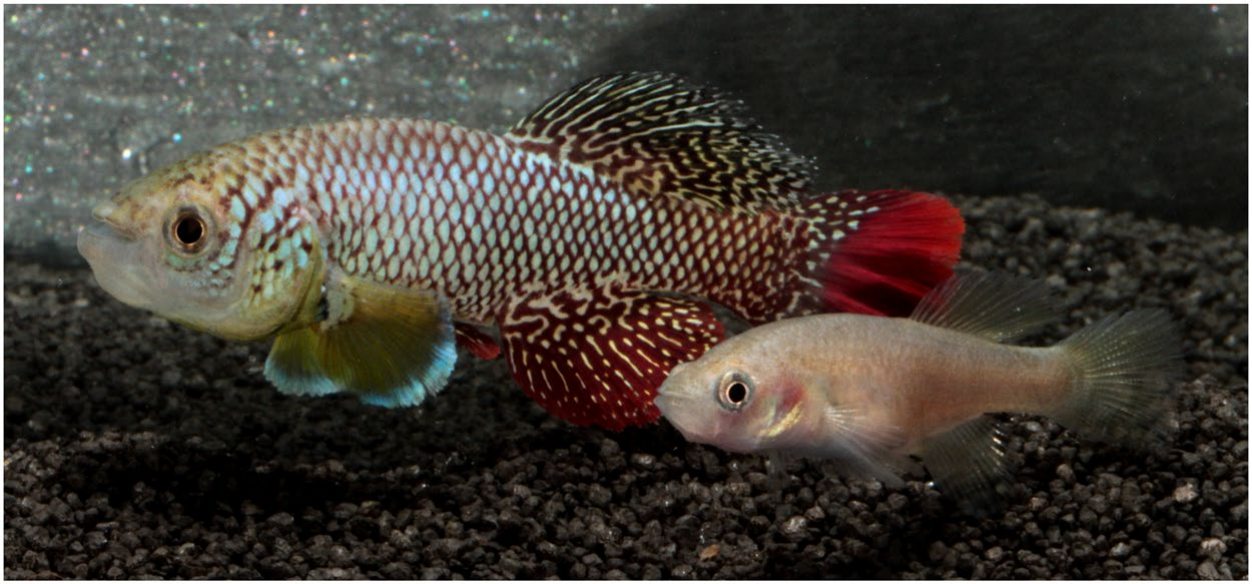
Male and female of turquoise killifish, *Nothobranchius furzeri*.

*Nothobranchius furzeri*, as with all other *Nothobranchius* species, are adapted to temporary pools that are formed during seasonal rains and desiccate early in the dry season^28^. Fish hatch from the eggs that survive the dry season in diapause, forming a single age cohort that inhabits the pool until its desiccation^29,30^. Dispersal among the individual pools is limited to years with exceptionally high precipitation and populations are strictly spatially separated in most generations^28,31,32^. In the laboratory, *N. furzeri* grow rapidly and mature in as little as 3 weeks under favourable conditions^15^. The sexes are markedly dimorphic with brightly coloured males and inconspicuous brown females (Fig. 1). Their sex-determination system is genetic, but sex chromosomes are not present^33^. The sex ratio is equal at the time of sexual maturity but many natural populations have female-biased adult sex ratios, likely a consequence of sexual selection (male-biased predation, higher male frailty or male-male competition)^18^. This indicates that while the ultimate disappearance of populations is caused by pool desiccation^14,17^, *N. furzeri* are subject to significant mortality throughout their lives. Identifying the sources of *Nothobranchius* spp. mortality in natural populations should provide critical insight for our understanding of the complex evolution of ageing.

Here, we present a longitudinal demographic study of wild populations of *N. furzeri* and their two sympatric congeners, *N. pienaari* and *N. orthonotus*. We followed numerous populations in southern Mozambique over a five-month period and examined the temporal dynamics in habitat conditions and killifish demographic (abundance, density, sex ratio) and community (species ratio) parameters. We predicted that population abundance would decline over the season while population density would increase due to the reduction in pool area and volume. We also predicted more rapid disappearance of males from the populations, leading to a female-biased sex ratio. Finally, we predicted a faster decline in the populations of *N. furzeri* compared to two sympatric *Nothobranchius* species, in accordance with the differences in their lifespans in a laboratory environment.

## Results

### Overview

We collected data for a total of 13 sites with up to three *Nothobranchius* species present (Table 1). Nine of those sites provided long-term demographic data. At two sites (LS3, LS4), the pools desiccated soon after the fish matured. One site (LS6) exhibited extremely low abundance of fish and another site (LN3) was too deep to support quantitative sampling. These 4 sites were used only in some of the planned analyses (for details see Table 1).

**Table 1:** Summary table for data obtained from the study sites.

Site	GPS coordinates	Species present	F clade	Hatching date	CPUE visits	Last CPUE	Final age	Analyses
Ch1	E 32°53′54″, S 22°16′33″	F, O	Chefu	26.1.16	13	25.5.16	120+	population size, density, sex ratio
Ch2	E 32°34′54″, S 22°30′28″	F, O, P	Chefu	26.1.16	1	11.3.16	72/−	sex ratio, species ratio
Ch3	E 32°43′38″, S 22°33′17″	F, O	Chefu	26.1.16*	3	5.3.16	45/−	population size, density, sex ratio, species ratio
LN1	E 32°34′01″, S 23°28′24″	F, O, P	Limpopo North	12.3.16	5	25.4.16	51/−	population size, density, sex ratio, species ratio
LN2	E 32°34′21″, S 23°30′14″	F, O	Limpopo North	12.3.16	—	NA	71/−	population size, sex ratio, species ratio
LN3	E 32°34′40″, S 23°31′47″	F, O	Limpopo North	10.3.16	—	NA	77+	sex ratio
LN4	E 32°36′01″, S 23°38′44″	F, O, P	Limpopo North	15.3.16	10	12.5.16	58/−	population size, density, sex ratio
LS1	E 32°22′45″, S 24°11′40″	F, O, P	Limpopo South	18.3.16	10	9.5.16	59/−	population size, density, sex ratio, species ratio
LS2	E 32°36'55″, S 24°18′15″	F, O, P	Limpopo South	10.1.16	5	21.4.16	110/−	population size, density, sex ratio, species ratio
LS3	E 32°44′33″, S 24°19′32″	F	Limpopo South	10.1.16*	—	NA	33/−	population size
LS4	E 32°41′37″, S 24°19′37″	F	Limpopo South	10.1.16*	—	NA	33/−	population size
LS5	E 32°46′30″, S 24°24′59″	F, P	Limpopo South	11.1.16	2	26.3.16	86/o	density, sex ratio
LS6	E 32°46′44″, S 24°25′08″	F, P	Limpopo South	10.1.16	—	NA	63/−	species ratio

**Figure 2:**
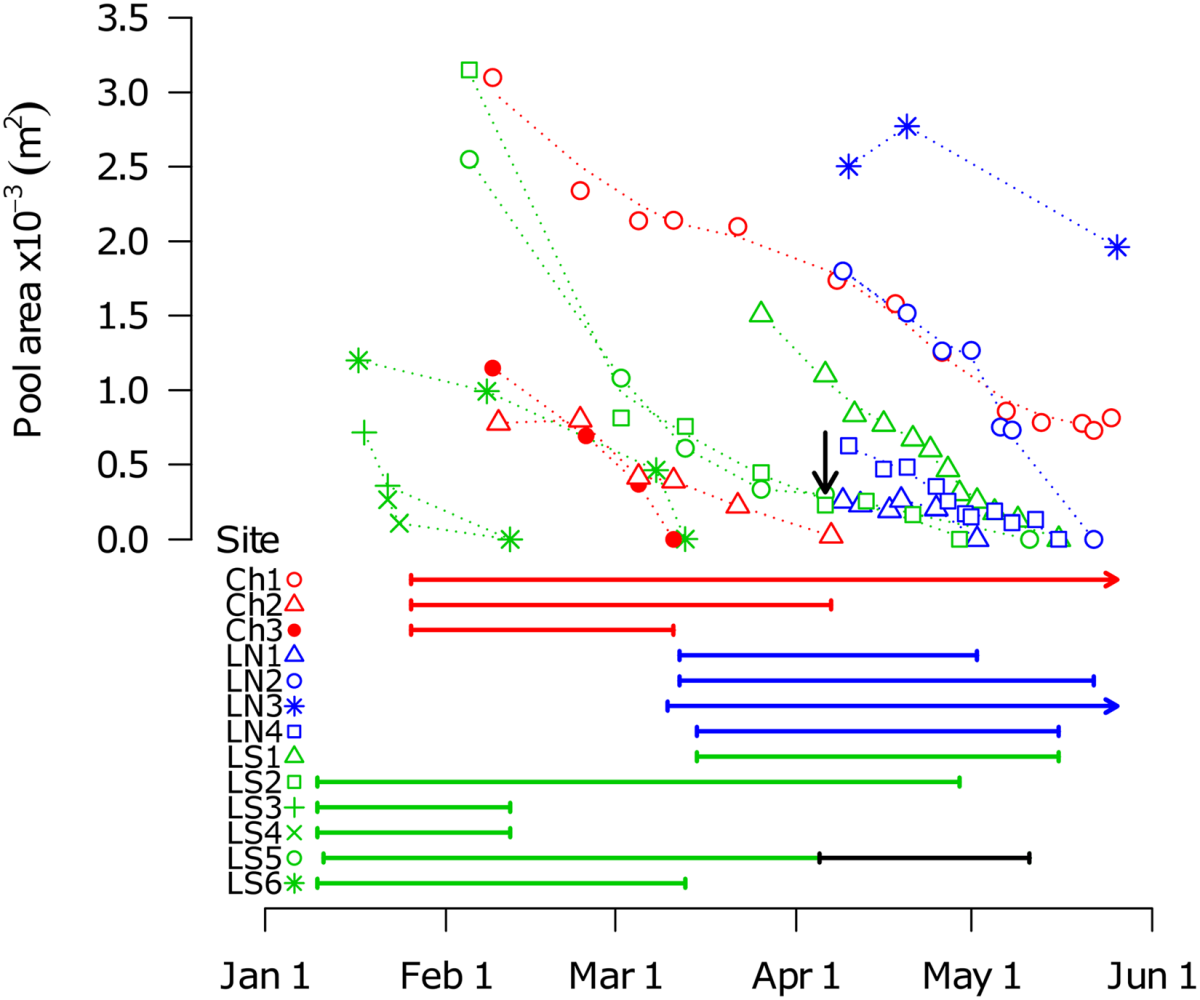
Pool area of the study sites throughout the 2016 rainy season. The points denote pool area when visited and the smooth dashed lines are fitted using LOESS function. The zero pool area marks the date when the pool was found to be dry. The black arrow shows a moment when fish disappeared from the pool LS5, while water was still present. The site-specific full lines show period between hatching and either pool desiccation, fish disappearance (site LS5, with the black line for period without fish), or termination of the fieldwork (arrowhead).

The abbreviation for *Nothobranchius* species present are F for *N. furzeri*, O for *N. orthonotus* and P for *N. pienaari*. F clade is phylogeographic lineage of *N. furzeri*^31^. Hatching date was estimated based on otolith readings and for sites, where not collected (*), the hatching date was estimated based on the hatching date estimates for fish in nearby sites (Supplementary Table 1). Final age is number of days the fish reached during our monitoring. The fish were present until pool desiccation (/−), fish disappeared, while the pool was still present (/o), or fish survived longer than the sites were monitored (+) (see also Fig. 2).

### Hatching date estimates

We estimated hatching date from readings of daily otolith increments for 10 sites (3 specimens per species per site) (Supplementary Table 1). There were three main periods of hatching across the study area, generally contingent upon division into three phylogeographic clades of *N. furzeri* (Table 1, Fig. 2). Fish from the Limpopo South and Chefu clades hatched in early and late January, respectively. The populations from Limpopo North hatched only after heavy late-season rains in mid March. The late-season hatching in site LS1 (Limpopo South) also corresponded with the rains in mid March.

### Population size and population density dynamics

The size of study pools was greatest immediately after filling and gradually decreased throughout the study period (Fig. 2). All but two pools desiccated before the end of our sampling (Fig. 2). The duration of existence of individual pools ranged from 33–110 days. One pool (site Ch1) persisted for more than 120 days (with *Nothobranchius* spp. present) when last visited on 25 May, as the fieldwork period was concluded. Another pool that still contained fish on 26 May (site LN3) had existed for 77 days at that time, as it was filled late in the rainy season.

Population size was estimated at 9 sites using either Catch-Per-Unit-Area (CPUA) or Capture-Mark-Recapture (CMR) estimates. Population estimates ranged over three orders of magnitude – from 53 individuals (*N. orthonotus* at LN2) to almost 20,000 individuals (*N. furzeri* at LS3) (Table 2). Repeated population size estimates, completed for two *N. furzeri* populations (LS2 and LS5), demonstrated that fish abundance declined sharply within a single month. A major decline to 14% of the original population size (LS5) was associated with a reduction of pool surface area to 42% of its original extent. The population decline to 47% of the original population size in the pool LS2 was accompanied by a reduction to 26% of pool surface area measured during the first CMR estimate (Table 2).

**Table 2:** Population-size estimates from Capture-Mark-Recapture (CMR) or Capture-per-Unit-Area depletion (CPUA) method. In the CMR, adult fish were marked with either visible implant elastomer tags (CMR-e) or by fin-clipping (CMR-f).

Site	Species	Method	Capture date	Area m^2^	Age	Captured fish	Recapture probability	Population size estimate (lower CI; upper CI)
Ch1	F	CMR-e	7.3.16	2138	41	145	0.19	748 (515; 981)
LN1	F	CMR-e	9.4.16	255	28	192	0.60	318 (264; 372)
LN2	F	CMR-e	19.4.16	1518	38	237	0.52	457 (402; 512)
—	O	CMR-e	19.4.16	1518	38	28	0.52	53 (31; 75)
LN4	F	CMR-e	10.4.16	626	26	210	0.15	1422 (1103; 1741)
LS1	F	CMR-e	11.4.16	836	24	74	0.36	204 (45; 363)
—	O	CMR-e	11.4.16	836	24	133	0.52	256 (189; 323)
LS2	F	CMR-f	5.2.16	3150	26	191	0.18	1054 (694; 1414)
—	—	—	2.3.16	812	52	41	0.08	492 (184; 800)
—	O	CMR-f	2.3.16	812	52	47	0.15	315 (135; 495)
—	P	CMR-f	2.3.16	812	52	126	0.11	1099 (694; 1504)
LS3	F	CPUA	23.1.16	356	13	877	0.34–0.50#	8714–19263~
LS4	F	CPUA	24.1.16	108	14	128	0.48–0.75#	577–1120~
LS5	F	CMR-f	6.2.16	2550	26	414	0.10	4247 (2827; 5667)
—	—	—	2.3.16	1081	51	140	0.23	613 (427; 799)

The population size in subadult fish was estimated only using enclosures and CPUA. The ranges of capture probability (#) and population size estimate (~) for CPUA are given.

**Figure 3:**
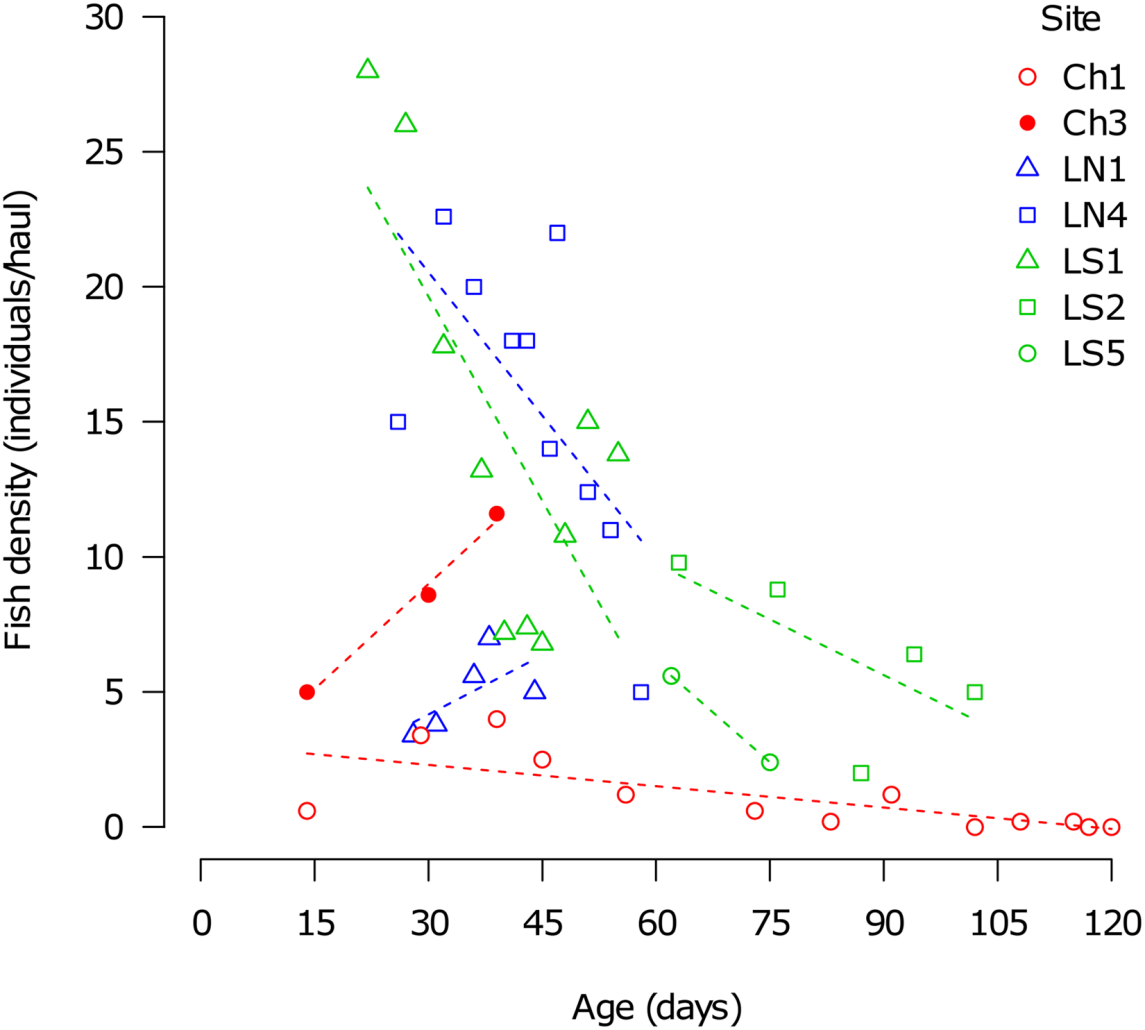
Temporal pattern of fish density at the study sites. Only sites with at least 3 visits with CPUE performed were analysed. The lines fitted represent predicted trend for each site from the generalised linear squares model (the estimated slope coefficients including their 95% CI are given in Supplementary Table 2).

Fish density was estimated as standardized Catch-Per-Unit-Effort (CPUE, the number of individual fish per net haul) at relatively regular intervals throughout the study period. An increase in fish density during the season was recorded only at a single site (Ch3), despite our prediction that density would increase as pool volume gradually shrank. Instead, fish density decreased significantly throughout the season in 3 out of 7 regularly sampled pools (3–13 visits per site) while no significant trend was detected in the remaining 3 pools (Fig. 3, Supplementary Table 2). Further, in two populations where CPUE indicated no decline in population density (CPUE), CMR sampling did detect a decrease (Table 2). Using the same approach, we additionally analysed density of *N. furzeri* separately from the other two species; the trends were comparable though weaker, as this analysis was based on a subset of data on all three species combined (Supplementary Fig. 1).

We were also unable to collect any *Nothobranchius* spp. despite substantial effort at one of the monitored, relatively small-sized, sites (LS5) 1 month before the pool actually desiccated (Table 1, Fig. 2). Collectively, this suggests that significant mortality is widespread among *Nothobranchius* spp. populations long before the pool desiccates.

### Seasonal dynamics in the adult sex ratio

**Figure 4:**
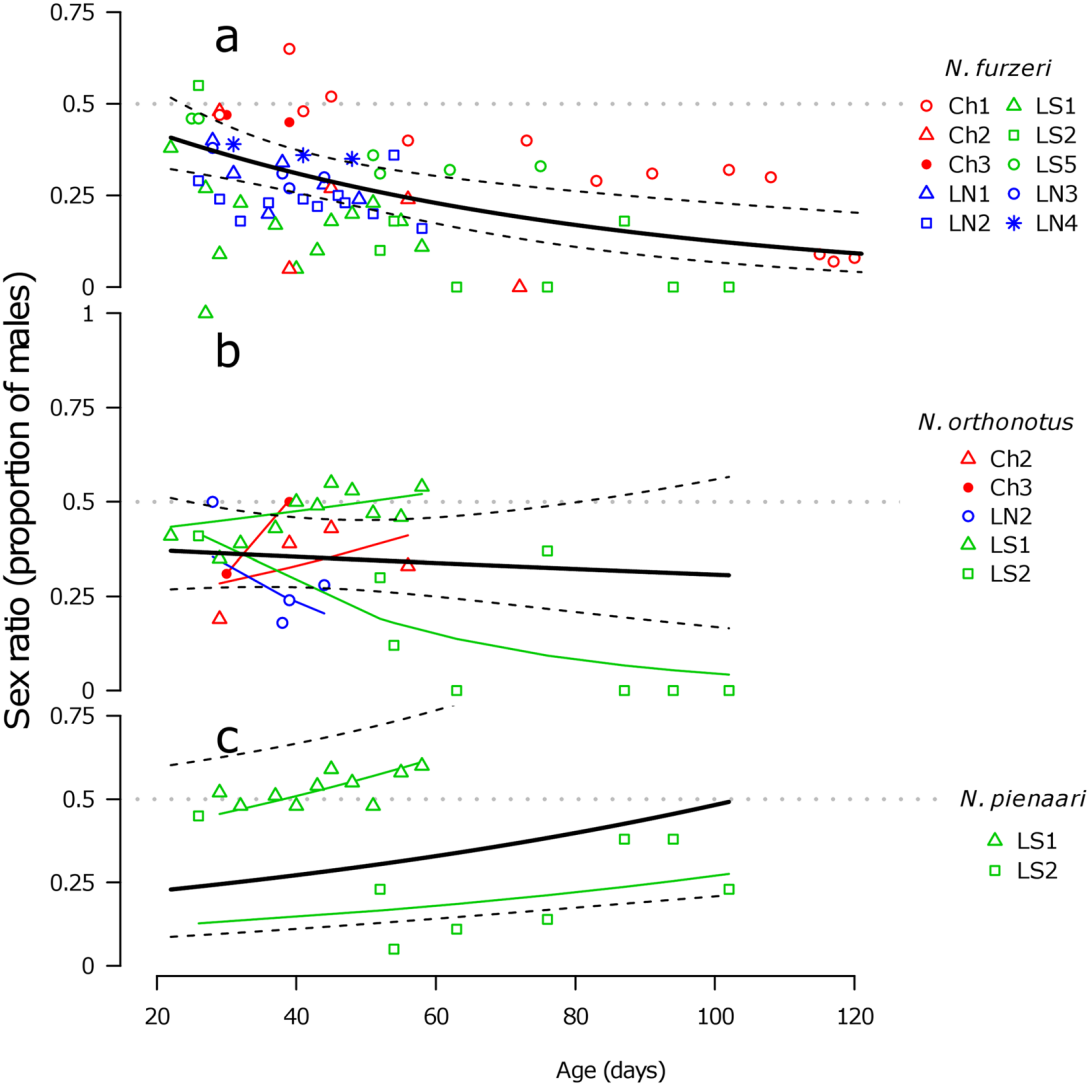
Temporal change in sex ratio. Seasonal change in sex ratio (proportion of males) in the three studied *Nothobranchius* species. In *N. furzeri* (**a**), the overall trend is negative, in *N. orthonotus* (**b**) inconclusive (population-specific) and in the two populations of *N. pienaari* (**c**), the proportion of males increased throughout the season. The thick black line gives overall trend estimated by the GLMM and dashed lines denote 95% CI.

We examined temporal dynamics in sex ratio as the proportion of males from the ‘Total catch’ (CPUE sampling complemented with additional sampling effort) in the three study species – *N. furzeri* (10 monitored populations, 70 sampling events, 7376 captured individuals), *N. orthonotus* (5 populations, 30 samplings, 1529 individuals) and *N. pienaari* (2 populations, 18 samplings, 1627 individuals). The seasonal trend in sex ratio varied among species (Fig. 4). The relative proportion of males declined in *N. furzeri* populations (GLMM: Estimate = −0.015, SE = 0.005, z-value = −3.190, P = 0.001, Fig. 4a). High interpopulation variability resulted in no overall trend in *N. orthonotus* (GLMM: Estimate = −0.006, SE = 0.006, z-value = −1.036, P = 0.300, Fig. 4b). The proportion of male *N. pienaari*, unexpectedly, increased throughout the season in both populations (GLMM: Estimate = 0.010, SE = 0.004, z-value = 2.221, P = 0.026, Fig. 4c), though a numerical male-bias was recorded only in one population (Fig. 4c).

### Species ratio

**Figure 5:**
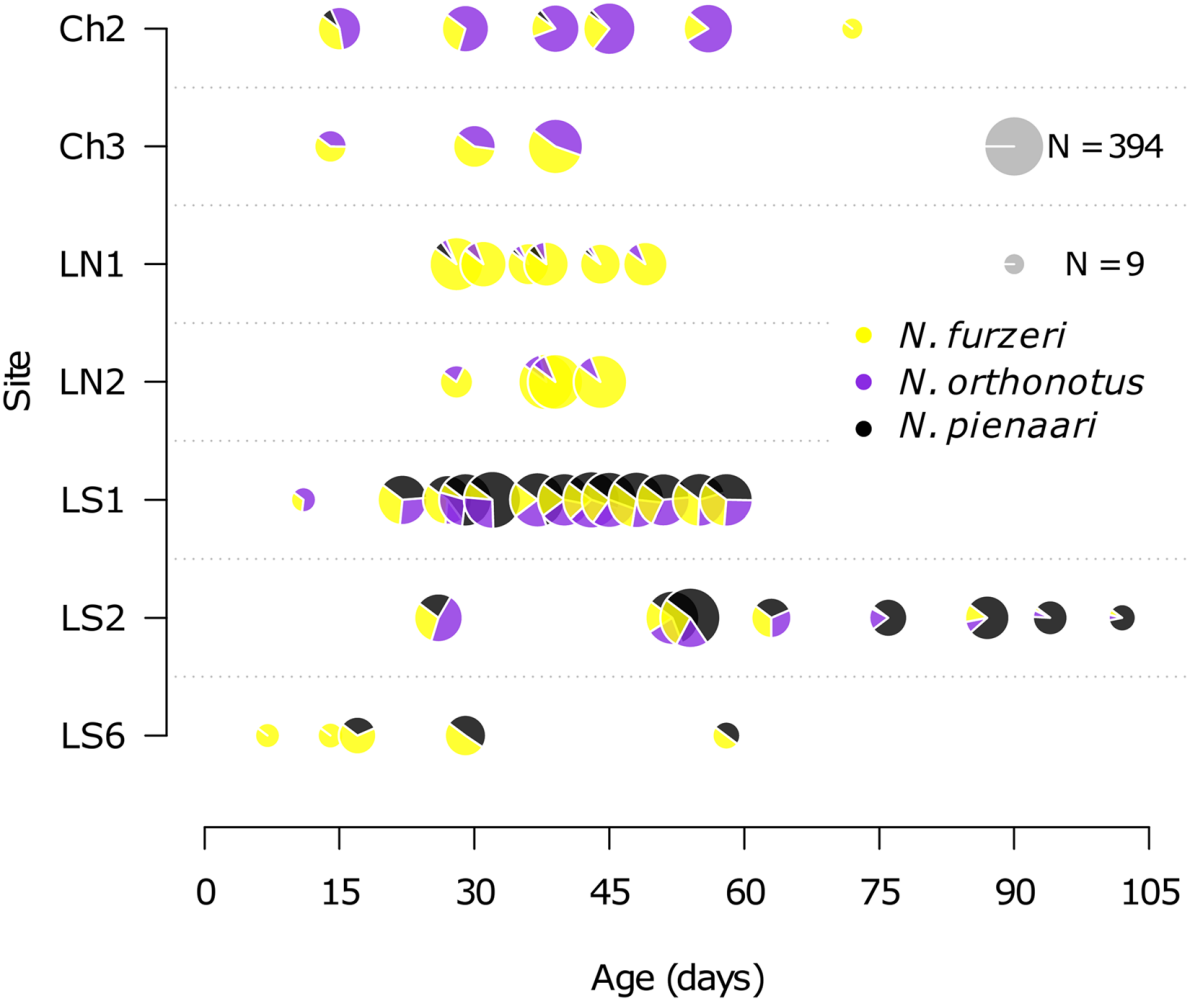
Proportions of up to 3 sympatric *Nothobranchius* species at 7 study sites throughout the season. The size of a pie gives number of fish in Total catch at each visit, thereby not necessarily showing population abundance. Note that only sites with 10 individuals captured per species at least once are included.

We performed 7 samplings at two sites where *N. furzeri* was sympatric with *N. orthonotus*, 5 samplings at a site where *N. furzeri* coexisted with *N. pienaari* (LS6), and 33 in four pools where all three species co-occurred. We did not record any non-annual teleost fish. In total, we collected 2476 individual *N. furzeri*, 1620 *N. orthonotus* and 1842 *N. pienaari*. The relative proportion of the three sympatric *Nothobranchius* species was site-specific (Fig. 5). *Nothobranchius furzeri* was present in all monitored pools, but its proportion in the community varied across pools. Neither the temporal pattern in abundance of *N. furzeri* (GLMM: effect of sympatry with *N. orthonotus* only – Estimate = 0.003, SE = 0.014, z-value = 0.198, P = 0.843; effect of sympatry with *N. pienaari* only – Estimate = −0.014, SE = 0.016, z-value = −0.860, P = 0.390; sympatry with both *N. orthonotus* and *N. pienaari* was used as an intercept), nor its relative abundance was affected by the presence or absence of particular coexisting species (GLMM: effect of sympatry with *N. orthonotus* only – Estimate = 0.707, SE = 0.386, z-value = 1.830, P = 0.067; effect of sympatry with *N. pienaari* only – Estimate = 0.430, SE = 0.550, z-value = 0.781, P = 0.435; sympatry with both *N. orthonotus* and *N. pienaari* was used as an intercept) and there was no overall seasonal trend in the proportion of *N. furzeri* in the *Nothobranchius* spp. community over the season (GLMM: Estimate = −0.003, SE = 0.005, z-value = −0.541, P = 0.589). There was no consistent pattern of temporal dynamics in species ratio and the sites were relatively stable in their community structure (Fig. 5).

## Discussion

The pools supporting killifish populations typically desiccated 1 to 4 months after filling but killifish sometimes disappeared from the pool well before the terminal habitat deterioration. We demonstrated that the density and abundance of *Nothobranchius* spp. populations declined considerably during the course of the season (Fig. 3, Table 2). This decline in population density contrasted with predicted increase in the population density due to gradual habitat shrinkage. Population size was in the order of hundreds to thousands of young adults (3.5–7.5 weeks old) but estimated to be up to an order of magnitude larger in subadults (2 weeks old fish). The sexes were subject to differential mortality but the seasonal dynamic in sex ratio was species-specific (Fig. 4). While male mortality was higher than female mortality in *N. furzeri*, sex bias in mortality was population-specific in *N. orthonotus*. In *N. pienaari*, the initial female bias was buffered later in the season. The three sympatric species co-existed at locally variable, but temporally relatively stable, proportions and *N. furzeri*, the shortest living species, did not disappear from the pools any earlier than the other two species.

The duration of the aquatic phase varied among pools, but with no relation to geographic location of the site (Fig. 2, Table 1). A previous study analysed data from visits to sites over multiple seasons (2011–2015) and demonstrated that pools in the drier part of the study area (Chefu region, Supplementary Fig. 2) desiccated significantly sooner than pools in the more humid Limpopo North region^14^. This outcome was consistently confirmed using independent datasets obtained from datalogger readings that detected desiccation as an abrupt change in ambient temperature amplitude^17,30^. In 2016, when the current study was conducted, there was a particularly light rainy season without the annual monsoon precipitation and intensive rainfall was regionally localised. We hypothesize that this masked the pronounced regional differences in the timing of pool desiccation observed in previous years. Nevertheless, our data demonstrate that the duration of the aquatic phase is variable among pools. Some populations survived only 33 days after hatching and almost half of the study sites (6 of 13) desiccated less than two months after filling (Table 1). In those pools, the main source of adult mortality was habitat desiccation. In contrast, study populations that persisted longer declined strongly before the pool desiccated, indicating that other factors, such as individual frailty to environmental and biotic challenges (e.g. high ambient temperature and low oxygen levels or aggressive encounters) or predation, were responsible for major mortality. Individuals from those populations were hence arguably subject to significant condition-dependent mortality (i.e. selective disappearance). Given that there is interannual variability in duration of specific pools^28^, the populations experience variable strength of random (pool disappearance) and selective (seasonal decline) mortality over successive generations^8^. This makes the selection pressure on annual killifish life history temporally dynamic, providing a potential explanation of the mismatch between selection on ageing and on other life history traits identified in an experimental study^14^. Under standardised laboratory conditions, lifespan and ageing varied predictably between populations from dry and wet regions, while reproductive allocation and behavioural and physiological aspects of the pace-of-life syndrome did not differ between contrasting populations^14^.

The limit imposed by pool existence is the ultimate selection force shaping annual killifish life history that is manifested by fast growth, early sexual maturation, high investment into reproduction and rapid ageing^8,14,15^. Naturally, a longer habitat duration provides more scope for condition-dependent mortality factors, as illustrated in Fig. 4 in ref.^8^. For example, both predation rate and predator richness are higher in temporary water bodies with extended duration^34,35^. High population density is known to facilitate the effect of condition-dependent factors on survival across various taxa^36–38^. Density-dependent regulation in *Nothobranchius* spp. may be further accentuated due to high levels of male aggression^14,39^ and their high *per capita* resource requirements^15,40^. We found support for this hypothesis as *Nothobranchius* spp. populations with high densities displayed the steepest density decreases (Fig. 3).

Individual marking and frequent sampling may potentially increase mortality of repeatedly sampled fish. We are confident that that our sampling regime did not cause significant mortality. We addressed this issue seriously at the stage of designing our sampling protocol as well as during the fieldwork. We only used a short distance haul (5 m) with the seine net to minimize negative impact of longer-term disturbance from net dragging. All collected fish were placed in a bucket with fresh water and strong aeration if not released immediately after capture. Both marking methods (finclipping, elastomer injection) have low impact on fish survival, as proven in previous pilot and published studies^14,18^. During elastomer tagging, that was more invasive marking technique and included anaesthesia, mortality of 0.5–2.5% was recorded. Finally, we documented no immediate mortality (presence of death fish) after returning the sampled fish back to the pool, and no delayed mortality after visit of the pool few hours after sampling (typically next morning).

The estimates of adult population size were an order of magnitude lower than for subadults. Population abundance for juveniles of 2 weeks of age was estimated as between 500 and 20,000 individuals using repeated depletions of enclosed sampling areas (CPUA estimate). The maximum estimated adult population size was always <5,000 individuals, using CMR methods. The use of different methods was imposed by logistic constraints; small juvenile fish could not be marked without compromising their condition and survival, while adult fish could not be enclosed in a netted area without a bias arising from disturbance and escape reaction. In fishes, juvenile mortality is typically enormous^41^ and we initially expected higher juvenile abundance than recorded. In many pools, we were unable to collect juvenile fish despite high sampling effort covering all available microhabitats. However, we collected adults in the same pools later in the season and their age estimates suggested that juvenile fish must have been present in the pools during our previous sampling events. Higher catchability of adult fish, perhaps partially explained by a decrease of pool surface area, is a possible reason for this discrepancy. We suggest that juvenile killifish mortality is relatively low compared to the typical mortality rate in juvenile teleost fishes^41^.

Male-biased mortality was clearly demonstrated in *N. furzeri*. Sex-ratio bias at the time of sexual maturation (20–40 days old) was small, but the proportion of males declined with age. At the age of 3 months, females always composed at least two thirds of the population and the sex ratio became even more biased in many populations (Fig. 4a). This is in agreement with results of Reichard *et al*.^18^, who reported consistently female-biased adult *N. furzeri* populations and a seasonal increase in female bias between early and late adult stages^18^. *Nothobranchius orthonotus* populations also tended to be female-biased, though only one population (out of 4) demonstrated a decline in male numbers over their lifespan (Fig. 4b). However, analysis in the other three populations was limited to a maximum age of 2 months. Overall population size declined sharply after that age, leaving us unable to draw firm conclusions concerning seasonal trends in the sex ratio of *N. orthonotus* populations.

Both populations of *N. pienaari* unexpectedly exhibited an increase in the proportion of males, oscillating close to an equal sex ratio in one population but with a persistent female bias in the second (Fig. 4c). The increase in the proportion of males in *N. pienaari* populations was unexpected^18^. *Nothobranchius pienaari* prefer the grassy microhabitat at pool margins and pilot sampling at site LS2 (strong initial female bias) showed that *N. pienaari* males were more likely than females to be found in the marginal vegetation. More specifically, over 50 dipnet sweeps in each microhabitat we found the sex ratio to be male-biased (14:9; males:females) in the vegetated littoral zone compared to a clear female bias (1:10) in the open water. Consequently, the apparent increase in female relative abundance may arise from different sampling efficiency in different habitats^42^ - females may have been more easily collected than males in the early stages of pool existence when dense grassy vegetation lined the pool margins. As gradual desiccation caused the water to recede from the vegetated pool margins, it is possible that males moved into the open water and were easier to capture.

Sex difference in lifespan and ageing is one of the unresolved phenomena of evolutionary biology and biogerontology. Males have a consistently shorter lifespan in many mammal species^43–45^ while females are the shorter living sex in most birds^46^. A female-biased sex ratio is common in populations of small fish where sexual selection plays a prominent role in male reproductive success, such as in Neotropical annual killifish^47^, Trinidadian guppies^42^, European bitterling^48^, as well as in other polygynous animals^46^. Whether this sex-bias in mortality is driven by selective predation on more conspicuous males^18,49^ or reflects intrinsic male frailty^50^ is currently not clear. While the presence of sex chromosomes^51^ is a plausible explanation for the contrast between mammals and birds, fish have diverse systems of sex determination and are a promising system for understanding the roles of sexual dimorphism in size, colouration and behaviour, and hormonal regulation of metabolism in sex-specific lifespan and ageing^52^.

At the interspecific level, we predicted that the proportion of *N. furzeri* in *Nothobranchius* spp. community would decrease over the season. Under benign laboratory conditions, *N. furzeri* is the shortest-lived species compared to its congeners^14,17^. While there was a trend towards selective disappearance of *N. furzeri* in some communities (Fig. 5: Ch2, LS2), the overall pattern was not statistically significant. We hypothesize that the natural lifespan of *N. furzeri* is relatively short compared to that observed in the laboratory and stronger changes in the species ratio would be observed in pools that persist longer than 2 months. Overall, *N. furzeri* is endemic to a relatively small region of the Limpopo and Chefu basins^32,53^, where conditions are more arid than in other parts of the *Nothobranchius* spp. range^8,17^. In this region, *N. furzeri* is the most common annual species, often coexisting with the geographically more widespread *N. orthonotus* and *N. pienaari*^53^. Reichard *et al*.^53^ suggested that the community pattern in species presence/absence is driven by abiotic factors rather than biotic interactions between *Nothobranchius* species and it is possible that *N. furzeri* is locally adapted to stronger fluctuation in habitat conditions compared to its sympatric congeners.

In conclusion, we demonstrated that natural *Nothobranchius* spp. populations declined strongly in abundance and were subjected to considerable mortality throughout the period of pool existence. The duration of aquatic phase varied across pools, with some populations experiencing only one month of post-hatching lifespan while others survived more than 4 months. Selective disappearance was apparent, with an increase in the sex-ratio bias in *N. furzeri* populations. Using several demographic measures, we demonstrated that the scope for selective mortality in *Nothobranchius* spp. populations before the pools desiccate can be substantial. Future research should address the functional aspects of condition-dependent mortality in wild populations of *Nothobranchius* species.

## Methods

### Study taxon

*Nothobranchius furzeri* is distributed throughout the semi-arid part of southern Mozambique and the adjacent part of south-eastern Zimbabwe^53^. Genetic analysis of wild populations revealed a relatively strong population genetic structure in *N. furzeri*, consisting of three major phylogeographic clades^31^. Populations from individual pools, often not more than a few kilometres apart, are significantly isolated^31,32^. Over much of its range, *N. furzeri* coexists with two congeneric species, *N. orthonotus* and *N. pienaari*^53,54^ that represent important comparative taxa^10,55^. *Nothobranchius orthonotus* and *N. pienaari* have a wider geographical distribution that extends to central Mozambique^53^. The three species exhibit subtle differences in their habitat preference^54^ and resource use^56,57^.

### Study sites

We performed longitudinal monitoring of 13 sites with *Nothobranchius* spp. between 17 January and 26 May 2016 (summarized in Table 1). The sites were distributed across the gradient in aridity (Supplementary Fig. 2) and rainfall predictability. More details on regional habitat conditions are provided elsewhere^18,53,54^. The sites were coded according to their affiliation to the *N. furzeri* geographic clade (Ch: Chefu region; LN: Limpopo North bank; LS: Limpopo South bank)^31^. Each site was visited between 3 and 14 times. We recorded pool area using a portable GPS device (Garmin GPSMAP® 64 s) by walking around the pool perimeter. The maximum depth of the pool was measured to the nearest 10 cm using a ruler. The presence of lungfish (*Protopterus* sp.), large predatory water bugs (Belostomatidae) and piscivorous birds was recorded (Supplementary Table 3) but their occurrence had no consistent geographic pattern.

### Age

The age of the fish was estimated from otolith samples collected at the monitored sites (Supplementary Table 1). Three individuals (mixed sex) per species per site were collected and stored in 96% ethanol. Otoliths were dissected, polished and the number of daily increments was read by a commercial facility (Barcelona Otolith Reading Services, Spain). The estimated hatching date was calculated by subtracting the age estimate from the date of collection. The earliest estimate is given where different ages were recorded to provide a population level commencement of the hatching period. Otolith samples were not available for three sites (Ch3, LS3 and LS4) so we estimated their hatching dates on the basis of those of adjacent sites where otoliths were read successfully (for Ch3 from Ch1 and Ch2, for LS3 and LS4 from LS2, LS5 and LS6; Table 1).

### Population density

We estimated Catch-per-Unit-Effort (CPUE) to compare fish density within pools between different sampling visits^58^. The CPUE typically consisted of 5 standardized hauls (5 m long) with a seine net (2.7 m long, 0.7 m deep, 4-mm mesh size), covering all habitats in the pool. Fewer hauls were completed when the pool was too small to avoid repeated sampling of the same area. The temporal dynamic in relative fish density was analysed for sites with at least three different CPUE sampling visits, providing data for 7 pools (Fig. 3).

### Population size estimate

We employed two separate methods (Capture-Mark-Recapture and Catch-per-Unit-Area) to estimate the size of *Nothobranchius* spp. populations at 9 sites (Table 2). The Petersen method of population size estimate by Capture-Mark-Recapture (CMR) was used^58^. We marked fish by clipping a small part of their caudal fin (2 sites) or by injecting visible implant elastomer tags (Northwestern Marine Technology, USA) (5 sites). The upper lobe of caudal fin was clipped during the first CMR estimate, while the lower lobe of the caudal fin was clipped during the second CMR estimate, conducted 4 weeks later. Fins of *Nothobranchius* spp. regenerate quickly^59^ and clipping from the first CMR estimate was almost undetectable after one month. Fish marked with elastomers were briefly anesthetized in a solution of clove oil and the tags were injected subcutaneously in two positions on their upper flank. Two marks were used to enable individual recognition for a separate study. After marking, fish were placed in fresh aerated water to recover and released back in the place of their capture. During marking and recovery, mortality was negligible (<2%). Fish marking was not size- or sex-selective; we marked all fish captured. The second sampling was conducted 15–24 h and 23–96 h after the first collection in populations marked using finclipping and elastomers, respectively. The entire pool area was always sampled, with the aim of collecting as many fish as possible over a 1–2 hour period.

The study pools were always isolated from other water bodies and migration was not possible. The Petersen formula for CMR population estimation: N = (C × M)/R was used, where N is the estimated population size, M is the number of fish captured and marked on the first sampling occasion, C is the number of fish captured on the second sampling occasion, and R is the number of individuals from the second sampling that had the mark. The 95% confidence intervals (CI) were calculated as N ± 1.96× standard error (SE), where SE = sqrt(M^2^ × C × (C − R)/R^3^)^60^.

Catch-per-Unit-Area (CPUA) is a method that extrapolates the catch from a part of the sampling area (typically an enclosure) to the entire sampling area. We used CPUA at two relatively small and shallow sites containing only *N. furzeri* (LS3, LS4; Table 2). Three square enclosures (2.5 × 2.5 m (6.25 m^2^) or 3 × 3 m (9 m^2^) for LS3 and LS4, respectively) were formed using a dense mesh (1-mm mesh size) supported by metal rods driven to the substrate at each corner of the square. Once the enclosure was set, fish were collected using short dipnet sweeps (triangular dipnet 45 × 45 × 50 cm, 2-mm mesh size) over a 5–15 min period, depending on fish abundance. These sampling periods were repeated until the number of fish captured reached approximately 10% of the fish abundance during the first sampling period.

We estimated the initial population size in the enclosure by calculating the depletion regression of sampling period catch on cumulative catch (Leslie method): C = q × N_0_ − q × ∑C, where C is the number of fish collected during each sampling session, q is catchability, N_0_ is the original population size and ∑C is the accumulated catch. The regression intercept equals q × N_0_ and the slope equals q^60^. The estimate from each enclosure (N_0_) was independently extrapolated to the entire pool area, providing repeated estimates of the total number of fish in the pool (range given in Table 2).

### Sex and species ratio

We employed additional sampling effort to the standard CPUE (pooled together as ‘Total catch’) to obtain a larger sample for estimates of population sex ratio and relative proportion of species present in the pool. We compared seasonal dynamics in sex ratio at 10 sites (17 populations of 3 species). Two rapidly desiccated sites (LS3, LS4) and one site with very low fish abundance (LS6) were excluded from the analysis. We also omitted sampling dates when juveniles were present, because small immature males cannot be reliably separated from adult females without dissection, providing potentially female-biased sex ratio estimates.

For the species ratio analysis, we selected sites that contained at least two species and where we collected at least 10 individuals per species on at least one sampling visit. This ensured that estimates were robust to random variation due to small sample size and enabled us to analyse *Nothobranchius* spp. communities from 7 sites, with 3–13 sampling events each (Fig. 5).

### Legal approval

All work was carried out in accordance with relevant guidelines and regulations. Sample collection complied with legal regulations of Mozambique (collection licence: ADNAP-170/7.10/16) and research procedures were approved by the ethical committee of the Institute of Vertebrate Biology, in accordance with legal regulations of the Czech Republic. No experimental work was carried out; therefore, no experimental protocols to be approved were available.

### Statistical analyses

All statistical procedures were conducted in the R environment (version 3.3.3, R Core Team). The response variables for respective analyses were Fish density, Sex ratio, and proportion of *N. furzeri* in the *Nothobranchius* spp. community. Explanatory variables were age (in days), site, species (for sex ratio analysis), level of sympatry (NF&NO, NF&NP, NF&NO&NP) (for proportion of *N. furzeri*). Data were not transformed. Defined models were visually inspected for any pattern in the distribution of residuals^61^ and tested for significance of the focal fixed terms (at level α = 0.05).

Fish density was the average number of *Nothobranchius* spp. individuals captured per haul in CPUE irrespective of species or sex. We fitted the data using generalised least squares models (GLS) with fish age, site identity and their interaction fitted as fixed factors. Different residual variance was allowed for each site.

Sex ratio was analysed as a bivariate variable - proportion of males caught per total catch (i.e. accounting for sample size) for a given species. We used binomial generalised linear mixed-effects models (GLMM) with logit-link function to test the effect of age on sex ratio for each species separately. We included both a random intercept (site) and slope (age) to account for repeated sampling. Similarly, the proportion of *N. furzeri* was a bivariate variable - the ratio of number of *N. furzeri* individuals to total fish caught at a site. A binomial GLMM with logit-link function was used to fit age and level of sympatry and both random intercept (site) and slope (age) were specified to correct for non-independence.

### Data availability

Original data are available at Figshare repository (10.6084/m9.figshare.5863431), with no delay after submission of the files.

## Electronic supplementary material

Supplementary information
