# Supplementary material for: Longitudinal demographic study of wild populations of African annual killifish
Source: Sci Rep. 2018 Mar 19;8:4774. doi: 10.1038/s41598-018-22878-6 (PMC5859278; doi:10.1038/s41598-018-22878-6)
Supplement: Supplementary file 1 — Supplementary information [file 41598_2018_22878_MOESM1_ESM.pdf]

Supplementary information

Longitudinal demographic study of wild populations of African annual killifish

Milan Vrtílek, Jakub Žák, Matej Polačik, Radim Blažek & Martin Reichard

---

Supplementary Figure 1. Map of distribution of the study sites along the precipitation gradient in southern Mozambique (yellow points – *Nothobranchius furzeri*, violet – *N. orthonotus*, black – *N. pienaari*). Dotted polygons show area of distribution of different phylogeographic clades of *N. furzeri* (Bartáková et al. 2013) (red – Chefu clade, blue – Limpopo North clade, green – Limpopo South clade). Rivers Chefu and Limpopo are illustrated by the black lines. Data on mean annual precipitation is based on 1960-1990 period and was obtained from www.worldclim.org.

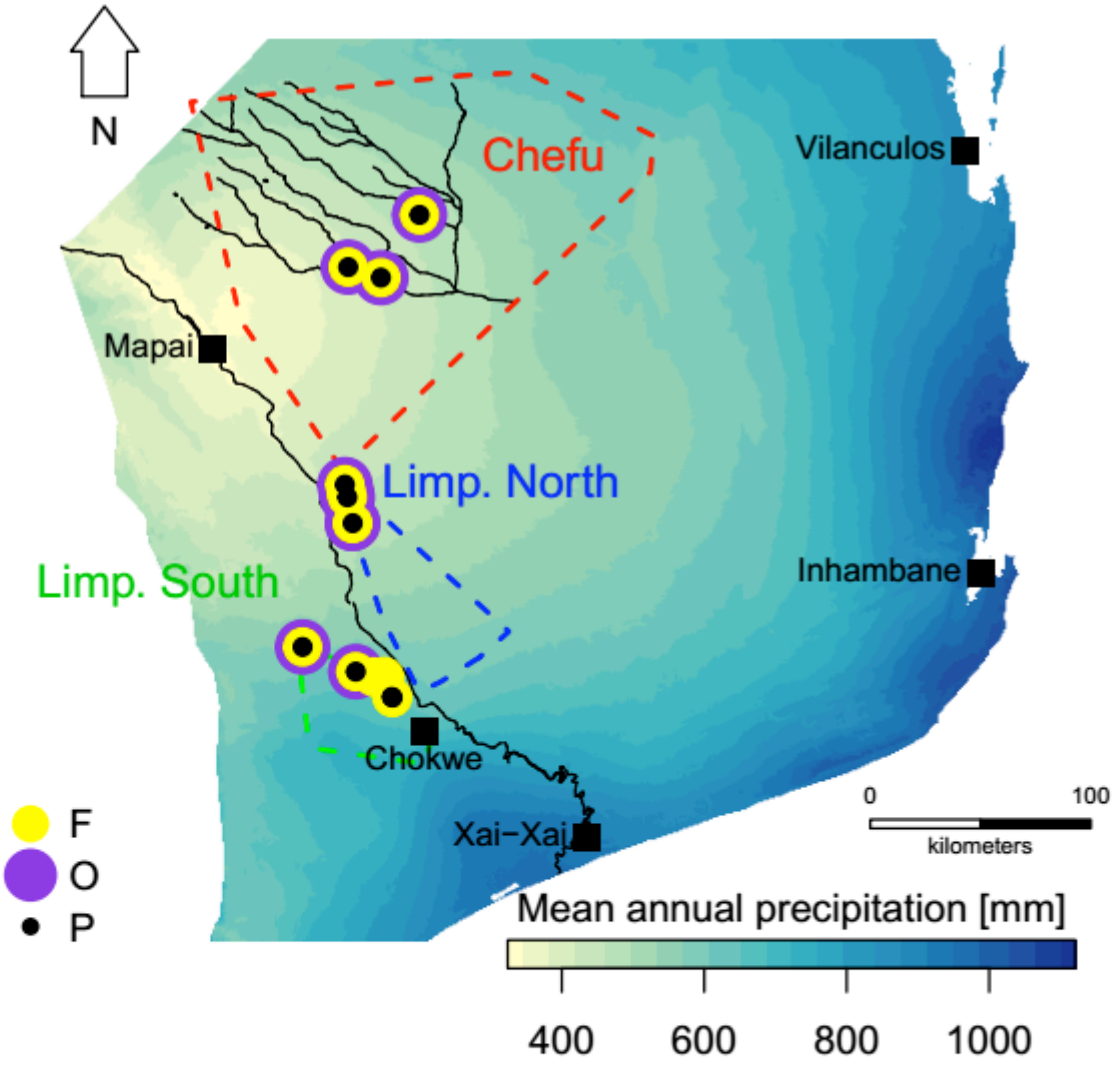

Supplementary Table 1. The estimates of age and hatching dates for the study sites from otoliths. Hatching dates in bold type-face were used in the analyses. The date of the first visit is given along the information, whether it is concordant with pool watering ('water').

ID	Site	F clade	Species	Sex	Collection date	Age estimate (2nd reading)	Hatching date	First visit
CZ-36	Ch1	Chefu	F	j	9.2.16	14	**26.1.16**	
CZ-37	Ch1	Chefu	F	j	9.2.16	14	26.1.16	9.2.16
CZ-38	Ch1	Chefu	F	j	9.2.16	14	26.1.16	
CZ-86	Ch2	Chefu	F	m	10.2.16	14(14)	**27.1.16**	
CZ-87	Ch2	Chefu	F	m	10.2.16	14(14)	27.1.16	
CZ-88	Ch2	Chefu	F	f	10.2.16	14(14)	27.1.16	10.2.16
CZ-83	Ch2	Chefu	O	m	10.2.16	14	27.1.16	
CZ-84	Ch2	Chefu	O	m	10.2.16	15(15)	**26.1.16**	
CZ-85	Ch2	Chefu	O	f	10.2.16	14	27.1.16	
CZ-18	LN1	Limpopo North	F	f	9.4.16	23	17.3.16	
CZ-19	LN1	Limpopo North	F	f	9.4.16	24(28)	16.3.16	4.3.16 water
CZ-20	LN1	Limpopo North	F	m	9.4.16	28	**12.3.16**	
CZ-53	LN2	Limpopo North	F	m	9.4.16	28(28)	**12.3.16**	
CZ-54	LN2	Limpopo North	F	m	9.4.16	28(28)	12.3.16	
CZ-55	LN2	Limpopo North	F	f	9.4.16	28(29)	12.3.16	
CZ-3	LN3	Limpopo North	F	f	10.4.16	36	5.3.16	
CZ-4	LN3	Limpopo North	F	m	10.4.16	31	**10.3.16**	10.3.2016 water
CZ-5	LN3	Limpopo North	F	m	10.4.16	28	**13.3.16**	
CZ-40	LN4	Limpopo North	F	m	10.4.16	20	21.3.16	10.3.2016
CZ-41	LN4	Limpopo North	F	f	10.4.16	26	**15.3.16**	water

CZ-42	LN4	Limpopo North	F	f	10.4.16	26(26)	15.3.16	
CZ-62	LS1	Limpopo South	F	j	26.3.16	abnormal otolith		
CZ-63	LS1	Limpopo South	F	j	26.3.16	7	19.3.16	26.3.16
CZ-64	LS1	Limpopo South	F	j	26.3.16	8	**18.3.16**	
CZ-9	LS2	Limpopo South	F	m	5.2.16	26	**10.1.16**	
CZ-10	LS2	Limpopo South	F	m	5.2.16	23	13.1.16	
CZ-11	LS2	Limpopo South	F	m	5.2.16	23	13.1.16	
CZ-74	LS2	Limpopo South	O	m	5.2.16	24	12.1.16	
CZ-75	LS2	Limpopo South	O	m	5.2.16	25(25)	**11.1.16**	5.2.16
CZ-76	LS2	Limpopo South	O	f	5.2.16	24(22)	12.1.16	
CZ-71	LS2	Limpopo South	P	m	5.2.16	24(24)	12.1.16	
CZ-72	LS2	Limpopo South	P	m	5.2.16	23	13.1.16	
CZ-73	LS2	Limpopo South	P	m	5.2.16	24(24)	12.1.16	
CZ-47	LS5	Limpopo South	F	f	5.2.16	24	12.1.16	
CZ-48	LS5	Limpopo South	F	m	5.2.16	25	**11.1.16**	5.2.16
CZ-49	LS5	Limpopo South	F	m	5.2.16	25	11.1.16	
CZ-65	LS6	Limpopo South	F	m	27.1.16	17(18)	**10.1.16**	
CZ-66	LS6	Limpopo South	F	m	27.1.16	17	10.1.16	
CZ-67	LS6	Limpopo South	F	f	27.1.16	16	11.1.16	17.1.16
CZ-68	LS6	Limpopo South	P	m	27.1.16	17(17)	10.1.16	
CZ-69	LS6	Limpopo South	P	j	27.1.16	17	10.1.16	
CZ-70	LS6	Limpopo South	P	j	27.1.16	17	10.1.16	

Supplementary Table 2. The sign of site-specific slopes from GLS model of fish density for the 7 study sites during the season (Fig. 2) including 95% CI. The interaction between site and age was used as explanatory variable. Correction for heteroscedasticity was set per site.

**Site**	**95% CI**	**Sign**
Ch1	-0.042; -0.011	-
Ch3	0.204; 0.315	+
LN1	-0.058; 0.350	0
LN4	-0.642; -0.067	-
LS1	-0.847; -0.161	-
LS2	-0.307; 0.031	0
LS5	-0.522; 0.030	0

Supplementary Table 3. Potential predators of annual killifish recorded at study sites during the 2016 rainy season. Water bug is large hemipteran sit-and-wait predator *Belostoma* sp., crab is *Potamonautes* sp. that opportunistically catch fish, lungfish *Protopterus annectens* is typically a night predator, and piscivorous bird category covers a range of species of herons, storks, hammerheads, kingfishers and terns that were observed at the sites and actively catch fish.

**Site**	**Water bug**	**Crab**	**Lungfish**	**Piscivorous bird**
Ch1	x	x	x	
Ch2		x		
Ch3	x			
LN1	x	x	x	
LN2	x	x	x	x
LN3	x	x		
LN4	x	x		x
LS1	x	x	x	x
LS2	x	x	x	x
LS3				x
LS4		x		
LS5	x	x	x	x
LS6	x	x		
